# Spatial Analysis of Health System Factors in Infectious Disease Management: Lessons Learned from the COVID-19 Pandemic in Korea

**DOI:** 10.3390/healthcare12151484

**Published:** 2024-07-26

**Authors:** Jeongwook Lee, SangA Lee

**Affiliations:** 1 Graduate School of Public Administration, Seoul National University, Seoul 08826, Republic of Korea; wookie@snu.ac.kr; 2 Manning College of Nursing and Health Sciences, University of Massachusetts Boston, Boston, MA 02125, USA

**Keywords:** infection outbreak, health system, public health, spatial dependence

## Abstract

Infectious disease outbreaks present ongoing and substantial challenges to health systems at local, national, and global levels, testing their preparedness, response capabilities, and resilience. This study aimed to identify and analyze critical health system-level factors that influence infection outbreaks, focusing on the experience of the COVID-19 pandemic in Korea. Conducted as a secondary data analysis, this study utilized national datasets from Korea. Given the inherent spatial dependencies in the spread of infectious diseases, we employed a spatial lag model to analyze data. While city-specific characteristics did not emerge as significant factors, health system variables, particularly the number of community health centers and health budgets, showed significant influence on the course of the COVID-19 outbreak, along with spatial autocorrelation coefficients. Our findings underscore the importance of enhancing public healthcare infrastructure, considering regional specificities, and promoting collaboration among local governments to bolster preparedness for future outbreaks. These insights are crucial for policymakers and healthcare professionals in formulating effective strategies to prevent, manage, and mitigate the impact of infectious disease outbreaks.

## 1. Introduction

Infection outbreaks pose persistent and formidable challenges to health systems globally, testing their preparedness, response capacity, and resilience [1]. The COVID-19 pandemic stands as a notable example, highlighting unprecedented challenges that led to widespread paralysis within healthcare systems. During this crisis, numerous healthcare professionals (HCPs) experienced physical and emotional burnout, hospitals were overwhelmed beyond capacity, academic institutions for HCP training were disrupted, and significant economic and human loss ensued [2,3]. Experiences from various infectious disease outbreaks, including Ebola, severe acute respiratory syndrome (SARS), Middle East respiratory syndrome (MERS), and, most recently, COVID-19, have provided critical lessons to the world regarding the functioning and adaptability of health systems at local, national, and global levels under crisis conditions [1,4,5].

Previous studies have shown that the risk of increasing infected cases is influenced by a range of individual demographic, environmental, and socioeconomic factors. Individuals’ knowledge, attitudes towards infectious diseases, and preventive behaviors are crucial [6]. For instance, during the COVID-19 pandemic, public compliance with health guidelines varied widely, influenced by how individuals responded to public health advisories and adhered to preventive measures such as vaccination, mask-wearing, and social distancing [7,8]. Additionally, environmental factors such as geographical location, climate, and urbanization have impacted the distribution of diseases [9,10].

However, the relationship between health system-level factors and infection outbreaks remains crucial yet inconclusive. The importance of health systems that are prepared against infectious outbreaks has been increasingly recognized [4]. A prepared health system encompasses overall readiness and response capabilities, the effectiveness of public health policies, the robustness of healthcare infrastructure, and socio-economic factors influencing health outcomes [1,4,5,11]. These systemic elements determine the scalability and sustainability of outbreak responses and are pivotal in managing widespread health crises effectively.

Therefore, this study aims to identify and analyze key health system-level factors that influence infection outbreaks, using the experience with COVID-19 in Korea. By examining these elements through the lens of the COVID-19 outbreak, we can identify gaps in the current health system, gain a comprehensive understanding of how systemic factors shape the course of infectious disease outbreaks, and propose evidence-based recommendations for strengthening the health system. The findings of this research will be of significant interest to policymakers, HCPs, and researchers in relevant fields. They will contribute to the ongoing efforts to improve health system preparedness and response capabilities, ensuring that the lessons learned from various infection outbreaks, including COVID-19, are effectively applied to future health challenges. 

## 2. Methods

### 2.1. Study Design and Data Sources

This study was a cross-sectional, secondary data analysis using the national datasets sourced from the Korea Disease Control and Prevention Agency (KDCA), the Korean Statistical Information Service (KOSIS), and the Korea Transport Database (KTDB). The KDCA [12] is a government agency responsible for developing and implementing disease control policies. KOSIS [13] is a national statistical database operated by Statistics Korea, which provides all nationally approved statistics gathered from more than 400 governmental agencies. KTDB [14] is a national transport database maintained by the Korea Transport Institute, which is a national research agency focused on developing transport policies and technologies.

The researchers focused on all city-level municipalities in Korea, utilizing cities as the primary unit of analysis. Korea has universal health coverage, and its cities share similar local government structures and relatively homogeneous demographic backgrounds. This context enables a more precise analysis of how health system factors impact the course of the infection outbreak. Data from all cities in Korea, except for remote islands, were collected, considering the spatial spread of the infection. A total of 225 cities were included for analysis in this study.

### 2.2. Variables

The main outcome variable is the cumulative number of confirmed COVID-19 cases per 100,000 people in a city. This measure is commonly used in epidemiology as it enables easier comparison across different cities, irrespective of population size [15]. The data on confirmed COVID-19 cases were obtained from the KDCA [12] by requesting the information through the Information Disclosure Portal operated by the Ministry of the Interior and Safety. Data collected from January 2020 to April 2021 were utilized to accurately capture the impact of health system variables. After this period, the national COVID-19 vaccination became more effective, with more than 10% of all citizens vaccinated, coinciding with a surge in COVID-19 cases across the country [16]. Therefore, to exclude the impacts of vaccination and the collapse of health systems resulting from the surge in cases, this specific period was chosen for analysis. 

Table 1 displays independent variables, their measurements, and the sources. The independent variables consist of health system factors and other characteristics of cities. The health system is defined as the people, institutions, and resources organized and aligned with established policies to enhance the health of the population they serve [17]. It compasses various components, including health service delivery, health workforce, health systems financing, health information systems, access to essential medicines, and leadership/governance [18]. This study specifically focused on health service delivery, workforce, and financing, as these are essential components of the health system and represent its immediate outputs. Given these components and the availability of data, this study examined health system factors such as the numbers of hospitals and community health centers (CHCs), health budget, the number of HCPs, and the number of emergency room (ER) beds, sourced from KOSIS [13]. The cities’ other characteristics encompass sex, age, educational level, single-person households, personal annual income, population density, foreigner population, social distancing, and CHC utilization, obtained from KOSIS [13]. Traffic volume data was obtained from KTDB [14].

Additionally, dummy variables for two regions (Seoul Capital Area and Daegu Metropolitan City) were created to control for exogenous effects. The Seoul Capital Area is home to half of the national population and is ranked as the fourth-largest metropolitan area in the world, harboring an extreme concentration of infrastructure and resources. The area consists of the largest and capital city, Seoul; the most populous province, Gyeonggi; and the second-largest metropolitan city, Incheon. Daegu Metropolitan City, the third-largest metropolitan city in Korea, comprising eight municipalities, experienced the initial explosion of COVID-19 cases primarily through religious centers at the onset of the pandemic. The distinct characteristics of these two regions highlight the need to consider their exceptional circumstances.

Given that the outcome variable collected between January 2020 and April 2021 was utilized, we used the independent variables from the year 2020. The independent variables were lagged by one year, meaning that the independent variables from the previous year were used to predict the outcome variable for the current year. These time-lagged independent variables helped to mitigate the issues of reverse causality and simultaneity, enabling the assessment of the persistent effects of independent variables on COVID-19 outcomes over time. All independent variables, except for CHC utilization, are based on 2020 data. The KDCA survey data on CHC utilization is from 2019 because the survey was temporarily suspended from 2020 to 2021 due to the COVID-19 pandemic.

### 2.3. Statistical Analysis

We examined the characteristics of the variables through descriptive statistics, analyzed the relationships between the variables using correlation analysis, and then performed regression analyses to identify factors influencing the outcome variable. Right-skewed variables such as health budget, personal annual income, traffic volume, and population density were log-transformed to reduce skewness, stabilize variance, and linearize relationships, thereby enhancing the accuracy and reliability of results in correlation and regression analyses.

Given the spreading nature of infectious diseases, they are likely to have spillover effects, also known as spatial dependence [19]. Spatial dependence refers to the process where the outcomes in some units influence the outcomes in other units, resulting in similar patterns among spatially proximate units [20]. Consequently, a spatial lag model (SLM), also known as a spatial autoregressive regression (SAR) model, was adopted to capture spillover effects in this study. The model is expressed as follows:

Y=ρWY+Xβ+ε

This model incorporates the spatial lag of the outcome variable, typically denoted as WY, where W is the spatial weights matrix and Y is the vector of observations of the outcome variable. This study employed a Queen contiguity-based spatial weight matrix, which is a commonly used method for defining spatial relationships. By incorporating WY, the model accounts for the influence that neighboring observations have on each other, effectively capturing spillover effects. This model is particularly useful for analyzing phenomena like the spread of COVID-19, where the outcome in one region is likely influenced by the outcomes in nearby regions.

Ordinary multiple regression models (OLS) assume independence between observations and do not account for spatial effects. However, in the presence of spatial dependence, estimates using traditional OLS are neither robust nor consistent [20,21,22]. Failure to account for spatial dependence can lead to a poor understanding of a social phenomenon [23], and the omission of spatial variables from a model can result in incorrect statistical inferences due to omitted variable bias. Therefore, it is necessary to adopt models that incorporate spatial dependence into the analysis. 

Spatial dependence can exist in either the dependent variable, the error term, or both, influencing the selection of an appropriate model. Specifically, the SLM addresses spatial dependence in the dependent variable, while the spatial error model (SEM) targets spatial dependence in the error term. The spatial autoregressive combined (SAC) model is designed to accommodate spatial dependence in both the dependent variable and the error term simultaneously. This study employed Lagrange multiplier (LM) tests to identify spatial dependence [24,25], which aids in selecting the most appropriate model among the three models. A significant LM lag statistic suggests using the SLM, a significant LM error statistic suggests using the SEM, and significant values for both suggest using the SAC model. In this study, the SLM was selected based on the significant LM lag statistic (*p* < 0.01) and the non-significant LM error statistic. This indicated that spatial dependence was present in the dependent variable, confirmed COVID-19 cases but not in the error term.

The results of the OLS model and the SLM were compared to determine the most suitable model for explaining the relationships between the infectious outbreak and influential factors. The variance inflation factor (VIF) of the variables included in the model indicated no multicollinearity issues. Statistical significance was established at a two-sided *p*-value < 0.05. All statistical analyses were performed using Python (v.3.12.4.).

### 2.4. Ethical Approval

The study used the secondary analysis of publicly available data; therefore, an ethics review process was not required [26]. Human subjects were not directly involved, and personal or private information were not prospectively collected and utilized without identifiers.

## 3. Results

### 3.1. Descriptive Statistics

Table 2 presents descriptive statistics for various variables across 225 cities. On average, there were 0.66 hospitals per 1000 people, with a range from 0.13 to 3.70. This variability indicated varying levels of healthcare accessibility and capacity across the studied cities. The mean of 0.23 CHCs per 1000 people underscored disparities in access to CHCs, ranging from cities with no centers to those with over one center per 1000 people. The average health budget was 14.97 million USD, with considerable variability ranging from 3.89 to 105.12 million USD across cities. With an average of 5.89 HCPs per 1000 people and a range from 0.94 to 50.36, disparities in healthcare workforce density were noticeable. The mean of 0.21 ER beds per 1000 people underscored varying levels of emergency care availability.

### 3.2. Geographical Distribution of Confirmed COVID-19 Cases

**Figure 1:**
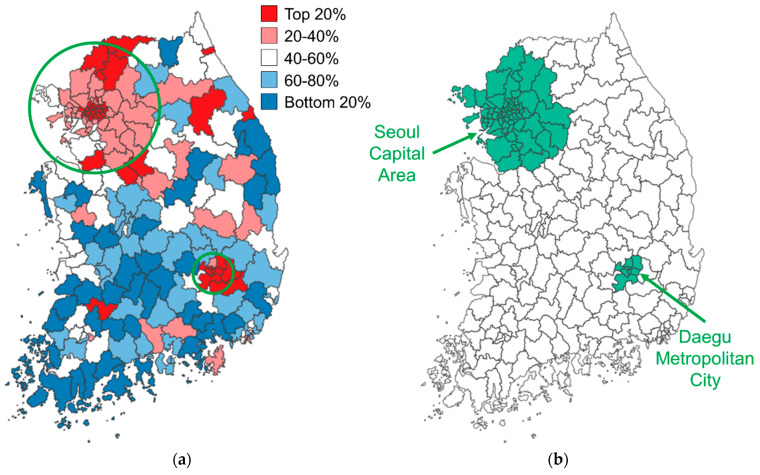
Geographical distribution of confirmed COVID-19 cases.

In Korea, the number of confirmed cases (per 100,000 people) across all cities averaged 199.66 ± 159.19, with a range from 9.58 to 1243.23 (Table 2). To further characterize the dependent variable, we examined the geographical distribution of confirmed cases from a spatial perspective. Figure 1a is a choropleth map depicting the geographical distribution of confirmed COVID-19 cases (per 100,000 people) based on five quantiles. Figure 1b shows the locations of the Seoul Capital Area and Daegu Metropolitan City. The number of confirmed cases was not evenly distributed across the country, with some cities showing clusters of high numbers of confirmed cases and others displaying clusters with low numbers. Notably, the Seoul Capital Area and Daegu Metropolitan City, colored green in Figure 1b, had the highest numbers of confirmed cases. Some cities neighboring the Seoul Capital Area and Daegu Metropolitan City also showed high numbers of confirmed cases. The clusters suggested that there was spatial dependence in the number of confirmed cases. This supported the need to incorporate spatial dependence into the model through spatial econometric methods.

### 3.3. Correlation Analysis Results

**Figure 2:**
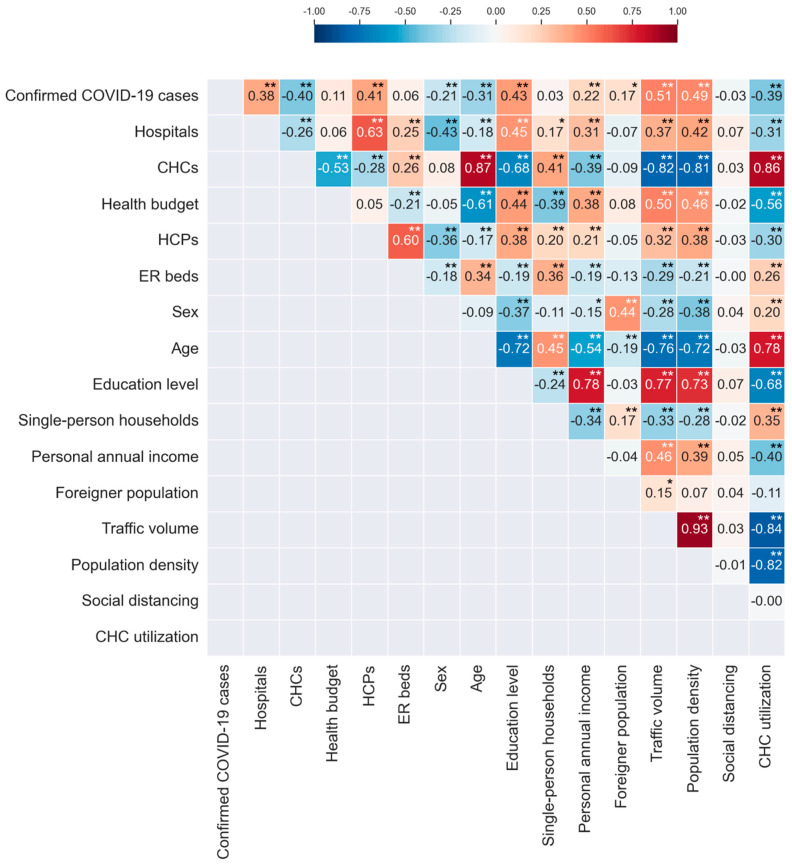
Results of correlation analysis. Note: CHC (community health center), HCPs (healthcare professionals), ER (emergency room). * *p* < 0.05, ** *p* < 0.01.

Figure 2 shows the relationship between confirmed COVID-19 cases (per 100,000 people) and other variables. Health system factors such as hospitals, CHCs, and HCPs exhibited stronger correlations in absolute terms with the number of cases compared to other factors such as sex, age, single-person households, foreigner population, and personal annual income.

Notably, among the health system factors, HCPs and hospitals showed significant positive correlations with the number of cases (0.41 and 0.38, respectively), while CHCs demonstrated a significant negative correlation (−0.39). Although health budget and ER beds also exhibited positive correlations with the number of cases, these relationships were not statistically significant. 

Among various city characteristics, variables significantly positively correlated with the number of confirmed cases, ranked by the magnitude of their correlation coefficients, included traffic volume, population density, education level, income, and the foreigner population. Conversely, variables significantly negatively correlated with the number of cases, ranked by the absolute values of their correlation coefficients, included CHC utilization, age, and sex.

### 3.4. Regression Results

The results of the non-spatial OLS model (Model 1) and the SLM (Model 2) are shown in Table 3. Model 2 is a spatial model with a spatial autocorrelation term that does not exist in the non-spatial Model 1. While the OLS model was a less complex model without a spatial term, several criteria suggest that the SLM was a more appropriate model. When evaluating statistical models, higher log likelihood and lower Akaike information criterion (AIC) and Bayesian information criterion (BIC) values collectively indicate that the model fits the data well while avoiding excessive complexity [27,28,29]. Firstly, the higher log likelihood of the SLM (−1360.825) compared to that of the OLS model (−1366.136) indicated a better fit. Moreover, the SLM boasted a lower AIC and a lower BIC (2759.649 and 2824.555, respectively) compared to those of the OLS model (2768.273 and 2829.763), suggesting it offered a better balance between model fit and complexity. The two models exhibit minor differences in empirical fit statistics, such as log likelihood and AIC, and four of the five significant variables appear in both models, which may raise statistical concerns. Previous studies have suggested both theoretical justification and data-based metrics for model selection [23,30]. The theoretical understanding is that disease spread is influenced by neighboring regions, which the OLS may not capture effectively. Therefore, the SLM was chosen for its better model fit, improved balance between model fit and complexity, and its strength to account for spatial relationships effectively in this study.

Of the diverse characteristics of cities, only single-person households were found to be significant in the OLS model, but no variables were significant in the SLM. Even the single-person households became insignificant in the SLM. This suggests that despite controlling for spatial effects, general city characteristics did not significantly influence the infection outbreak. 

Regional dummies for the Seoul Capital Area and Daegu Metropolitan City showed significance in both the OLS and SLM. The significance of such regional dummies in both models suggested that these cities had unique circumstances significantly impacting COVID-19 cases. Both cities are major urban centers in Korea, and their significance in both models could reflect higher population densities, extensive transportation networks, or other factors contributing to the spread of COVID-19. Additionally, municipalities within Daegu Metropolitan City experienced a significant number of confirmed cases concentrated in religious facilities, which the regional dummy variable effectively captured to represent these unique characteristics of the area.

Notably, the spatial autocorrelation coefficient for COVID-19 cases in the SLM was highly significant, underscoring the necessity of including spatial variables in the model. The coefficient (ρ) in the SLM represents the strength and direction of spatial autocorrelation among observations. A positive coefficient indicates spatial autocorrelation, suggesting that areas with higher numbers of COVID-19 cases tend to be surrounded by areas with similarly high numbers of cases. Specifically, a one-unit increase in the COVID-19 cases in a city is associated with an increase of approximately 0.293 COVID-19 cases in neighboring cities, holding other variables constant.

Most importantly, when accounting for spatial effects of the SLM, certain health system factors were found to be statistically significant. The number of CHCs and health budget showed significance in both the OLS and SLM. The SLM model exhibited lower absolute coefficients, indicating a potential overestimation in the OLS model. In the SLM, an increase of one CHC per 100,000 people was associated with a decrease of approximately 141 cases per 100,000 people. A 1% increase in the health budget size (before log transformation) corresponded to a decrease of approximately 0.368 cases per 100,000 people. This relationship was derived from the coefficient estimated in the regression model, where Δ *Y* ≈ *β* × Δ ln(Health budget) = *β* × ln(1.01) = −36.967 × 0.00995 = −0.368. While the health budget showed a positive but non-significant correlation with the number of confirmed COVID-19 cases in the correlation analysis, its regression coefficient was negative and statistically significant (*p* < 0.05). The significance of CHCs and the health budget in both the OLS and SLM models underscores the notion that cities with a greater number of CHCs or allocated higher health budgets are likely better equipped to navigate the complexities of the COVID-19 pandemic. This could contribute to decreased case counts and lower risks for infection outbreaks.

## 4. Discussion

This secondary data analysis study explored the impact of health system factors on the COVID-19 pandemic using national datasets from Korea. Unlike previous studies that primarily focused on individual- or population-level factors, this research investigated health system-level factors such as healthcare facilities, HCPs, and health budgets of cities and spatial relationships with neighboring cities. The study found that the number of CHCs and health budgets significantly influenced the COVID-19 outbreak dynamics, emphasizing their role in pandemic response strategies. Based on these findings, several recommendations can be proposed for policymakers, HCPs, and researchers in related fields to enhance infection outbreak response strategies. 

This study enhanced the accuracy of its findings by employing statistical analyses that accounted for the infectious nature of COVID-19, including the use of regional dummy variables and the SLM. In the literature on infectious diseases, few studies have integrated these spatial effects into their analyses. Most studies have focused on mapping the distribution of disease burden, encompassing factors such as incidence, prevalence, and mortality [31,32,33]. Meanwhile, researchers in other fields examined spatial spillover effects. Examples include economic policies affecting neighboring economies [34,35], the spread of pollutants and invasive species in environmental science [36,37], policy diffusion in public administration [38,39], crime patterns in criminal justice [40,41], and transportation impacts in urban planning [42,43]. These studies illustrated how interconnected geographical regions influence diverse phenomena, underscoring the interdisciplinary nature of spatial spillover research. The COVID-19 pandemic has demonstrated that outbreaks transcend national borders or administrative boundaries, highlighting the importance of analyzing spatial relationships to make precise predictions and facilitate rapid and proactive responses.

Our dataset, derived from official national datasets of Korea, exhibited considerable variability across many studied variables. This high variability can be partially attributed to Korea’s unique demographic and geographic distribution. Approximately 50% of Korea’s population resides in the Seoul Capital Area, which occupies only 25% of the country’s total land area. This dense urban population contrasts sharply with the less densely populated rural areas, leading to significant variability in confirmed cases, healthcare access, resource allocation, and infection rates. Previous research on other infectious diseases has also reported high variability within Korea [44]. Additionally, studies from the United States, Germany, Colombia, and Korea have documented similar high variability in key health system factors, such as hospital beds, ICU beds, and primary care physicians [45,46,47,48].

The final SLM model with controlling for cities’ characteristics suggests that the number of CHCs and health budgets are crucial in mitigating the COVID-19 outbreak. While CHCs was a significant influential factor, hospitals was not, likely due to their differing roles. Hospitals focus on patient treatment, whereas CHCs emphasize prevention and public health management. It underscores the importance of improving public healthcare infrastructure and increasing financial resources to enhance pandemic preparedness and response. As widely recognized, CHCs are vital for primary healthcare, particularly in underserved areas [49]. Their roles encompass clinical services, community resource coordination, and health coaching and education [50]. Despite their frontline role, there is limited investigation into enhancing CHCs and the preparedness of HCPs working within them for infection outbreaks. Most studies conducted in community settings have focused on the impacts of infection outbreaks on the field, highlighting the challenges faced by HCPs during outbreaks [51,52] rather than their preparedness and proactive practices. Drawing lessons from the COVID-19 pandemic underscores the importance of the prevention and preparation of infection. Therefore, specific strategies are needed to enhance CHC capabilities, including optimizing their number and location, to effectively and efficiently address community health needs in future outbreaks. 

Furthermore, it is widely recognized that health budgets play a crucial role in supporting CHCs and other healthcare facilities. While the existing literature predominantly focused on the economic burden of infections, the discussion should pivot towards how national and local government health budgets are structured and are utilized to effectively respond to infectious diseases [53]. Specifically concerning CHCs, adequate financial resources enable them to maintain operational readiness, procure essential medical supplies, allocate HCPs, and implement proactive infection control measures. Therefore, enhanced investment in CHCs and increased health budgets should be integral components of preparedness and response frameworks for future pandemics and infectious disease outbreaks.

Managing CHCs and budgets falls under local government jurisdiction. Therefore, local authorities should clearly define their roles and perspectives. Given that infectious diseases can spread rapidly across regions, regardless of administrative boundaries, cooperation among neighboring regions is crucial. However, existing studies often focus on individual cities or hospitals, with limited evidence on inter-city or CHC cooperation. Previous studies on non-infectious diseases [54,55] have demonstrated successful city-to-city and CHC-to-CHC collaborations. According to Swann et al. [54], interorganizational collaboration by local governments positively correlated with their capacity to provide treatment for opioid use disorder, with this connection being more pronounced in rural communities than in metropolitan ones. Langabeer et al. [55] examined how interorganizational collaboration among healthcare organizations influenced treatment times for acute myocardial infarctions. Collaboration among neighboring regions or CHCs in sharing resources, information, and best practices holds substantial promise for enhancing pandemic preparedness and response, leading to better resource allocation, quicker response times, and consistent public health measures. Further investigation into such collaborative strategies among neighboring regions or CHCs is essential for effective disease control and comprehensive pandemic preparedness. 

This study has several strengths that enhance both internal and external validity. Conducted in Korea, with its universal health coverage and demographic homogeneity, this study offers unique advantages for studying infectious diseases. The reduced need to control for ethnic, racial, and cultural variables enhances internal validity and allows for simpler statistical models, leading to clearer interpretations and more robust conclusions. By minimizing confounding effects, researchers can better understand the factors driving disease transmission and the effectiveness of public health measures. Additionally, the study utilized national datasets from Korea, providing a comprehensive overview that captures regional variations for holistic analysis. This broad scope enhances the external validity of the findings, ensuring generalizability across different regions and contexts within the country. Combined with the spatial spillover effects model, these strengths enhance the study’s ability to accurately analyze and comprehend the spread of COVID-19. The consistent significance of variables across both OLS and SLM models reinforces the robustness of these findings. It indicates that the observed relationships with COVID-19 cases are not contingent on specific modeling techniques but are genuine across various analytical approaches. This consistency instills confidence among policymakers and public health experts in utilizing these results to formulate effective strategies to mitigate the pandemic.

The study has several limitations. Firstly, as a cross-sectional study, it does not capture the temporal dynamics and evolution of disease spread over time. Secondly, the focus on the early stages of the COVID-19 pandemic means that the findings may not reflect the later stages or long-term effects. A more comprehensive approach using a panel data model that spans all stages of the pandemic would provide a deeper understanding of how various factors influenced its course over time. Incorporating panel data would strengthen the findings’ robustness and offer more practical insights for future public health strategies. Additionally, while hospitalized patients, intensive care cases, and home ward cases are significant in the literature [56,57,58], the limitations of the available data prevented their inclusion in this study’s analysis. Lastly, as this study focused on the quantitative aspects of health systems, it did not address their qualitative dimensions. Further research could incorporate quality aspects to gain deeper insights into how they influence health outcomes during infectious disease outbreaks.

## 5. Conclusions

This comprehensive analysis aimed to enhance our understanding of health systems in managing infectious outbreaks and to guide the development of robust, scalable, and sustainable health policies for future threats. In conclusion, our findings highlight the critical roles of CHCs, health budgets, and spatial effects in influencing COVID-19 case counts. Policymakers, HCPs, and researchers should consider these factors when designing and implementing public health interventions aimed at effectively preventing, managing, and mitigating the spread of infectious diseases. Improving public healthcare, considering regional characteristics, and fostering collaboration among local governments are essential strategies in the fight against infectious outbreaks. 

## Figures and Tables

**Table 1:** List of variables and their measurements.

Variables	Measurements	Sources
Confirmed COVID-19 cases	Cumulative number of confirmed COVID-19 cases per 100,000 people in a city from January 2020 to April 2021	KDCA [12]
Hospitals	Number of hospitals in a city per 1000 people	KOSIS [13]
CHCs	Number of community health centers in a city per 1000 people	KOSIS [13]
Health budget	Budget amount (converted to millions of USD) allocated to the health sector by a city government ^†^	KOSIS [13]
HCPs	Number of medical doctors and nurses in a city per 1000 people	KOSIS [13]
ER beds	Number of emergency room beds in a city per 1000 people	KOSIS [13]
Sex	Male population divided by the female population, multiplied by 100, in a city	KOSIS [13]
Age	Median age of city residents	KOSIS [13]
Educational level	Percentage of city residents with a bachelor’s degree or higher	KOSIS [13]
Single-person households	Percentage of single-person households in a city	KOSIS [13]
Personal annual income	Income per worker reported (converted to thousands of USD) during the year-end tax filing for earned income in a city ^†^	KOSIS [13]
Foreigner population	Percentage of foreigners living in a city at the time of survey who have lived in Korea for more than 3 months, relative to the total city population	KOSIS [13]
Traffic volume	Estimated number of vehicles (cars, buses and trucks) travelling along roads within a city per day	KTDB [14]
Population density	Persons living in a city per km^2^	KOSIS [13]
Social distancing	Percentage of city residents who practiced social distancing in the past week at the time of survey	KOSIS [13]
CHC utilization	Percentage of city residents who used community health centers in the past year at the time of survey	KOSIS [13]

Note: CHC (community health center), HCPs (healthcare professionals), ER (emergency room), KDCA (Korea Disease Control and Prevention Agency), KOSIS (Korean Statistical Information Service), KTDB (Korea Transport Database). ^†^ 1 USD = 1200 KRW (Korean Won).

**Table 2:** Descriptive statistics.

Variable	Count	Mean	Standard Deviation	Minimum	Maximum
Confirmed COVID-19 cases (per 100,000 people)	225	199.66	159.19	9.58	1243.23
Hospitals (per 1000 people)	225	0.66	0.36	0.13	3.70
CHCs (per 1000 people)	225	0.23	0.29	0.00	1.23
Health budget (Million USD) ^†^	225	14.97	10.38	3.89	105.12
HCPs (per 1000 people)	225	5.89	6.04	0.94	50.36
ER beds (per 1000 people)	225	0.21	0.16	0.00	1.11
Sex	225	100.92	6.28	88.00	124.80
Age (years)	225	46.09	4.83	36.90	57.40
Educational level (%)	225	26.24	10.09	11.50	62.75
Single-person households (%)	225	33.48	5.06	18.26	51.91
Personal annual income (Thousand USD) ^†^	225	29.70	5.50	21.15	62.09
Foreigner population (%)	225	3.09	2.29	0.51	12.94
Traffic volume (vehicles/day)	225	6272.99	4980.03	788.00	22,335.00
Population density (persons/km^2^)	225	3856.49	6006.79	18.31	25,225.51
Social distancing (%)	225	95.35	4.61	67.20	99.90
CHC utilization (%)	225	31.10	12.46	13.10	67.80

Note: CHC (community health center), HCPs (healthcare professionals), ER (emergency room). ^†^ 1 USD = 1200 KRW (Korean Won).

**Table 3:** Results of the OLS Model and the SLM.

	Model 1 (OLS)			Model 2 (SLM)		
Variable	Coefficient	SE	*p*	Coefficient	SE	*p*
Spatial Autocorrelation Coefficient for COVID-19 Cases (ρ)				0.293	0.079	0.000 **
Hospitals	6.521	30.319	0.830	10.024	28.159	0.722
CHCs	−177.399	73.340	0.016 *	−140.806	68.334	0.039 *
Health budget	−44.517	18.568	0.017 *	−36.967	17.333	0.033 *
HCPs	4.324	2.356	0.068	3.765	2.190	0.086
ER beds	54.143	79.222	0.495	73.261	73.603	0.320
Sex	1.653	2.127	0.438	1.191	1.978	0.547
Age	4.453	3.468	0.201	2.853	3.246	0.379
Educational level	0.788	2.209	0.722	0.188	2.058	0.927
Single-person households	4.177	2.094	0.047 *	3.106	1.952	0.112
Personal annual income	46.619	95.008	0.624	35.393	88.239	0.688
Foreigner population	3.361	4.564	0.462	3.051	4.243	0.472
Traffic volume	25.873	30.270	0.394	19.207	28.119	0.495
Population density	5.327	10.713	0.620	4.311	10.089	0.669
Social distancing	−1.677	1.651	0.311	−2.302	1.534	0.133
CHC utilization	2.179	1.361	0.111	1.965	1.266	0.121
Seoul Capital Area	180.980	25.005	0.000 **	137.451	25.604	0.000 **
Daegu Metropolitan City	300.299	43.431	0.000 **	226.423	44.492	0.000 **
Constant	−612.624	544.290	0.262	−345.673	506.731	0.495

Note: CHC (community health center), HCPs (healthcare professionals), ER (emergency room). * *p* < 0.05, ** *p* < 0.01.

## Data Availability

Dataset available on request from the authors.
